# The health-related quality of life, mental health and mental illnesses of patients with inclusion body myositis (IBM): results of a mixed methods systematic review

**DOI:** 10.1186/s13023-022-02382-x

**Published:** 2022-06-16

**Authors:** Katja C. Senn, Laura Gumbert, Simone Thiele, Sabine Krause, Maggie C. Walter, Klaus H. Nagels

**Affiliations:** 1 Chair of Healthcare Management and Health Services Research, University of Bayreuth, Parsifalstrasse 25, 95445 Bayreuth, Germany; 2 SMA Europe, Im Moos 4, 79112 Freiburg, Germany; 3 Friedrich-Baur-Institute, Department of Neurology, Ludwig-Maximilians-University of Munich, Ziemssenstrasse 1, 80336 Munich, Germany

**Keywords:** Health-related quality of life, Inclusion body myositis, Neuromuscular diseases, Mental health, Rare diseases

## Abstract

**Background:**

Inclusion body myositis (IBM) is a rare neuromuscular disease (NMD) and effective therapies are not available. Thus, it is relevant to determine the health-related quality of life (HRQoL) in IBM patients including aspects of mental health and illnesses.

**Objectives:**

To identify and summarize the assessment of HRQoL, mental health and illnesses in IBM, the major factors that determine and influence them as well as the respective influence of IBM in general and compared to other NMD as a systematic review.

**Methods:**

We performed a mixed methods systematic review according to the Preferred Reporting Items for Systematic Reviews and Meta-Analyses (PRISMA) guidelines. The search was conducted within the databases PubMed, PsycINFO, LIVIVO and the Cochrane Database. Data were narratively summarized and categorized in the physical, psychological and social HRQoL dimensions.

**Results:**

The systematic screening totalled 896 articles. Six studies were finally identified, comprising of 586 IBM patients. The applied patient reported outcome measures (PROMs) varied. Quantitatively, the main physical impairments (e.g. weakness, functioning, role perception) were assessed using the general population or other NMD as comparators. Results on social and psychological HRQoL were frequently inconsistent. Qualitatively, psychological and social limitations accompanied IBM related physical deteriorations.

**Conclusions:**

A research gap exists regarding rigour determinants of HRQoL and mental illness in IBM. In-depth qualitative studies could help to prepare the ground for the assessment of long-term HRQoL data combined with appropriately focussed psychological PROMs advancing the understanding of the HRQoL in IBM throughout the course of the disease from a patient perspective.

**Supplementary Information:**

The online version contains supplementary material available at 10.1186/s13023-022-02382-x.

## Background

Inclusion body myositis (IBM) is a slowly progressive idiopathic inflammatory muscle disease (IIM). Up to 50% of patients are wheelchair-bound after a 14-year disease duration [1, 2]. Prevalence ranges between 4.5 and 9.5 per million, and up to 139 per million in elderly populations over 50 years [3–5]. The frequently asymmetric muscle weakness first affects the quadriceps femoris or finger flexors. 40% of patients additionally report mild swallowing problems at the time of IBM diagnosis, increasing up to 80% of patients in the course of the disease [6–8]. To date, causative treatment is not available, and IBM frequently does not respond to treatment [9].

Previous research hast indicated that neuromuscular diseases (NMD) primarily impact the physical dimension of health-related quality of life (HRQoL). Insights on their impact on the psychological and social parameters are rather sparse [10, 11]. Moreover, mental or psychiatric comorbidities occurring simultaneously with somatic disorders are often overlooked in patients with noncommunicable diseases [12, 13]. In 40% of somatic patients, anxiety or depression disorders occur during lifetime, suggesting a prevalence twice as high compared to the general population [14].

Numerous studies on HRQoL have been undertaken across heterogeneous clinical phenotypes in NMD in the past. Reviews of HRQoL in NMD often fail to outline comparable values of the HRQoL in differing NMD. There is a paucity of evidence to demonstrate extensive between group variations of HRQoL in NMD [10, 11, 15, 16]. A systematic review published in 2016 underlined the neglected research intensity in the past: only two empirically backed-up studies in IBM patients were identified [17]. Accordingly, there is a high need to gain a comprehensive understanding of the disease trajectory and HRQoL in IBM. Although etiology and new therapeutic approaches become a wider research field [9, 18, 19], the assessment of HRQoL could further enhance patient-centric decision making in clinical practice to identify and select the best care option in the light of finite healthcare resources.

Therefore, we conducted a mixed methods systematic review to conceptualize the stipulated holistic understanding of HRQoL and the role of determinants of mental health and mental illnesses in IBM [10, 20, 21]. We aimed at answering the following research questions:

(i) How are the HRQoL and especially mental health and mental illnesses assessed in IBM patients?
(ii) To what extent does IBM influence the dimensions of HRQoL, especially mental health and mental illness in general and compared to other NMD?
(iii) Which determinants influence HRQoL of IBM patients and how can they be assessed?

## Methods

We followed the PRISMA 2020 checklist [22, 23] for our pre-defined systematic review protocol, registered at PROSPERO database (#CRD42020182072). Since individual patient data were not collected, compliance with data protection regulation was fulfilled and an ethical approval was not necessary.

### Eligibility criteria

The focused context of the included studies was based on the recommendations of PROGRESS-Plus [24], PRISMA Equity Extension [25] and CICI Framework [26]. The following inclusion criteria were applied: (1) language: English or German; (2) peer-reviewed qualitative or quantitative articles, not classified as a review or meta-analysis; (3) outcomes/perspective: HRQoL, measured with generic or disease-specific patient reported outcome measures (PROMs) or qualitative studies aiming to describe HRQoL dimensions and determinants; (4) IBM patients; (5) no filters for publication date. Studies were excluded if they: (1) examined other NMD, without disaggregated outcomes for IBM; (2) evaluated primarily interventions or (3) epidemiological outcomes; (4) only assessed distinct symptoms, complications or single dimensions of HRQoL; (5) were not reported as peer-reviewed articles; (6) were animal or (7) clinical or genetic studies. These specific criteria are supposed to prevent drawing analogies from the results of other NMD to IBM patients and thus increase the internal validity. We focused an explorative historical design for our systematic review. As the latest established diagnostic criteria for IBM were published after the year 2010, our aim was to identify all studies with individuals named IBM patients to give a comprehensive overview of the actual care situation from the past until now.

### Search strategy

The search was performed on 11 February 2021 using the Medline (via PubMed), PsycINFO (via Ovid®), LIVIVO and Cochrane databases. It was supplemented with a hand search via Google Scholar and screenings of bibliography. If the required full-text data were missing, the authors or study investigators were personally contacted. Keywords and MeSH-Terms for “IBM”, “HRQoL”, “mental health” and “mental illness” were combined and adapted to the syntax of the respective databases. Additional file 1 provides the detailed search strategies. KS started the development process of the search strategy with identifying keywords, synonyms and thesaurus terms as MeSH terms for Medline. The InterTASC ISSG for the specific study focus “Quality of life” was used to validate search terms [27]. The process was peer reviewed by LG and KN, who are experienced in systematic review searches in health economics and health services research. According to the eligibility criteria, filters for humans as well as for English and German language were applied.

### Selection process

Titles, abstracts and full texts were screened for eligibility by two independent reviewers (KS, LG).

### Data extraction process and data items

The following data items were extracted from the included studies: setting, number of study participants (= *n*), distribution of gender, age, IBM diagnostic criteria, duration and age at onset of the disease, disease severity and reported symptoms, outcome measures, main results and conclusions. The qualitative data extraction followed the GRADE-CERQual approach [28]. The data items were extracted in a predefined grid (KS) and checked (LG) independently. Disagreements were resolved by consensus.

### Quality assessment

The risk of bias assessment was conducted for the included cohort studies with the Newcastle–Ottawa scale (NOS) [29]. Qualitative studies were assessed with GRADE-CERQual [28], which also considers a possible meta-bias. To evaluate the overall quality of cross-sectional studies, the Appraisal tool for Cross-Sectional Studies (AXIS tool) [30] was used. The Mixed Methods Appraisal Tool (MMAT) [31] was applied additionally to efficiently summarize a quality assessment for all studies. Discrepancies after assessment (KS, LG) were resolved by discussion.

### Data synthesis

As IBM is a rare disease, we expected a small specific body of evidence with a low evidence level. Therefore, an exploratory mixed methods approach and a narrative synthesis with “weaving” technique [32, 33] was applied.

## Results

### Selected studies

**Fig. 1:**
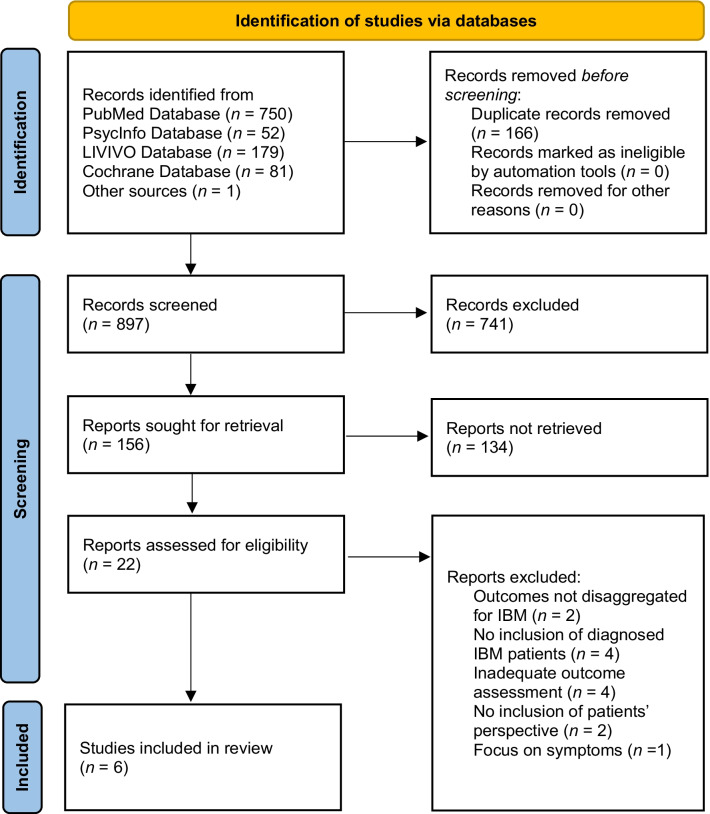
Flowchart of the screening process according to PRISMA 2020 [23]

The systematic search identified 896 titles after removing duplicates. 156 abstracts and 22 full texts were screened. One additional article was found along with a hand search. Two excluded studies did not disaggregate baseline HRQoL outcomes for IBM, focusing either on influences of muscle density in IIM (primary research paper, IBM *n* = 5) or focusing assessment instruments for disease activity and damage in IIM (non-primary report). Finally, six studies were included [34–39]. Figure 1 illustrates the selection process.

### Basic study characteristics

The basic characteristics of the included articles are shown in Table 1. All six studies were conducted in high-income countries: USA [35, 36, 39], USA and Canada [34], Australia [37] and the UK [38]. Patients were recruited either via patient registries [34], specialized neuromuscular clinics [34–36, 39], calls for participation on websites of professional societies, [34] or from pre-existing studies [37, 38]. All articles were published between 2010 and 2017. Four studies had a cross-sectional design [34, 36, 38, 39], whereas one analysed RCT data [38]. Two studies employed qualitative methods [35, 37].

**Table 1:** Basic characteristics and quality rating of the included studies

Study, year	Context country	Study design	Study population total/N	Female IBM *n*/%	Applied diagnostic criteria IBM n	Risk of bias assessment		
						NOS	AXIS	MMAT
Feldon et al. [34], 2017	USA, Canada	Cross-sectional	IIM 1648/ PM 481 DM 702 IBM 465	186/40	*n* not stated; possible or probable IBM [40];*	7/9	Yes: 11 No: 5 Do not know: 4	Yes: 5 No: 0 Can’t tell: 0
Goyal et al. [36], 2016	USA	Cross-sectional	IBM 25	6/24	19 clinically defined IBM, 6 probable IBM [42]	5/9	Yes: 12 No: 3 Do not know: 5	Yes: 2 No: 1 Can’t tell: 2
Rose et al. [39], 2012	USA	Cross-sectional	NMD 302/ LGMD 91 FSHD 49 PM/DM 19 IBM 24 MD 79 Misc 40	6/25	> 6 months with confirmed diagnosis**	7/9	Yes: 14 No: 2 Do not know: 4	Yes: 4 No: 0 Can’t tell: 1
Sadjadi et al. [38], 2010	UK	Cross-sectional (RCT data)	IBM 60	22/37	*n* not stated; definite or possible IBM [40]	6/9	Yes: 8 No: 4 Do not know: 8	Yes: 4 No: 0 Can’t tell: 1
Gibson et al. [35], 2016	USA	Qualitative	IBM 10	4/40	10 clinicopathologically defined IBM [42]	–	–	Yes: 5 No: 0 Can’t tell: 0
Ortega et al. [37], 2010	Australia	Qualitative	IIM 14/ PM 8 DM 4 IBM 2	not stated	*n* not stated; applied criteria [41] “South Australian Database for Patients With Biopsy-Proven Inflammatory Myositis”	–	–	Yes: 1 No: 1 Can’t tell: 3

AXIS: Appraisal tool for Cross-Sectional Studies; DM: dermatomyositis; FSHD: facioscapulohumeral muscular dystrophy; IBM: inclusion body myositis; IIM: idiopathic inflammatory myopathies; IQR: interquartile range; LGMD: limb girdle muscular dystrophies; MD: myotonic dystrophy; MMAT: Mixed Methods Appraisal Tool; NMD: neuromuscular diseases; NOS: Newcastle–Ottawa scale; PM: polymyositis

*Additional validation of patients’ self-reports with a partial sample (6.7% of N), 87% matching with a physician’s diagnose

**Muscle biopsy, genetics, raised creatine kinase levels, neurophysiology, expert opinion

#### Study population and applied diagnostic criteria

This review summarizes findings from 586 IBM patients. The mean sample size in the cross-sectional studies was 143.5 (24–465). On average, the proportion of female participants was 33.2% (24–40%). The qualitative studies included either two [37] or ten [35] IBM patients. Some studies included only IBM patients [35, 36, 38], others examined IBM in the context of IIM with polymyositis (PM) and dermatomyositis (DM) [34, 37] or with other NMD [39].

The applied IBM diagnostic criteria followed either Griggs et al. [34, 38, 40] or the European Neuromuscular Centre [35–37]. Two studies did not specify the diagnostic criteria but mentioned databases with biopsy-proven diagnosis for IIM [41] or an expert based IBM diagnostic [39].

#### Risk of bias assessment

Table 1 presents the identified rather high risk of bias. The following adjustments or considerations should be noted. Sadjadi et al. [38] used basic data of RCTs and was therefore treated as a cross-sectional study, due to the lack of follow-up data and the different study objective in contrast to the RCTs.

In accordance with the aim of this review, item 1 of **NOS** was interpreted, whether HRQoL was recorded as a PROM. Thereby, no stars were assessed in the selection domain ‘Endpoint Pre-Existence’ and outcome domain ‘Missing Data’ for all studies. Relating to **AXIS**, a “No” was rated for item 3 ‘Justification of Sample Size’ and “Do not know” for item 5 ‘Selection of Sample Size’ and ‘Representation of Target Population’. Item 14 ‘Information about Non-Responders’ was three times rated with “No” and once with “Do not know”.

Table 3 comprises the summary of our qualitative findings according to **GRADE CERQual** and Additional file 2 comprises the respective evidence profiles. The confidence in the extracted qualitative findings was either “moderate” and “low”, or “low” and “very low”.

The consolidated bias assessment with the **MMAT** showed most varying values for item 4.2 ‘Representation of Target Population’ and item 4.4 ‘Risk of Nonresponse Bias’. Overall, the MMAT showed minor differences to the specific tools.

### Description of the study populations

Table 2 shows a detailed description of the included patients. The **age** (years) of the included IBM patients was reported either as range (47–85), mean (64.47, 58.1) or median (67, 70) [35, 36, 38, 39]. NMD patient groups ranged from 34 to 76, mean 63 ± 11.6 [37, 39].

**Table 2:** Overview of study populations and research aims of the included studies

Study	Age IBM patients* years	Age at onset/diagnosis* years	Disease Duration* years	Disease Severity* IBM specific	Disease severity measures** Others	Research Aims Regarding HRQoL and Mental Health/Illness
Feldon et al. [34]	Not stated;	Not stated/Median 62.3 (IQR 55.5–68.2)	Median 9.2 (IQR 5.3–13.6)	Not stated	Questionnaire with disease-related information	HRQoL in adult IIM compared to RA and normal population, predictors of lower HRQoL in IIM
Goyal et al. [36]	Median seropositive 67 (47–77), seronegative 70 (60–85)	Median seropositive: 55.5 (45–71), seronegative: 54.0 (54–78)/not stated	Median seropositive:10.0 (3–15), seronegative: 11.0 (4–24)	*IBMFRS* median seropositive: 23 (17–36), seronegative: 29 (22–35), (p = 0.06)	6 min walk test, timed get up and stand test, MRC, right and left hand grip, NIF, pressure meter, mRS, mOBFRS	Exploring HRQoL according to NT5c1A antibody
Rose et al. [39]	Mean 63 ± 11.6	Not stated	> 0.6	Not stated	HAQ	Impact of chronic muscle disease upon HRQoL, exploring disease severity, mood and illness perceptions upon HRQoL
Sadjadi et al. [38]	Mean 64.47 ± 8.47	Not stated	Mean 4.35 ± 2.96 (0–10.8)	Not stated	ALS-FRS, MMT, QMT	Impact of IBM upon HRQoL, impact of disease severity upon HRQoL, identification of alternative assessments for IBM relating to HRQoL, impact of depression on relationship of disease severity and HRQoL
Gibson et al. [35]	Mean 58.1 (50–80)	Not stated	Not stated	Not stated	Not stated	Impact of pertinent symptoms upon HRQOL and daily functions
Ortega et al. [37]	Not stated; total study population (34–76)	Not stated	Not stated	Not stated	Not stated	Patients’ areas of concerns and impact of myositis upon daily living to discuss with rheumatologists

ALS-FRS: Amyotrophic Lateral Sclerosis Functional Rating Scale; HAQ: Health Assessment Questionnaire; IBMFRS: IBM functional rating scale; MMT: manual muscle testing; mOBFRS: modified oral bulbar facial respiratory scale [46]; MRC: Medical Research Council score; mRS: modified Rankin Scale; NIF: negative inspiratory force; QMT: qualitative muscle testing;

*Values are reported as median, mean ± SD, (range) or (IQR)

**Due to the aim of the description of disease specific HRQoL, the values for non-specific disease severity were not extracted in detail

**Disease onset** referred to the age at onset (median 55.5 and 54) [36] or to the age at diagnosis with a median of 62.3 [34], where IBM patients were significantly older at diagnosis in contrast to PM and DM, in line with the typical clinical presentation (both p < 0.0001).

Three articles covered information on the **disease duration** in years as mean (4.35) or median (10 and 11) [34, 36, 38]. Rose et al. [39] included patients living with IBM > 0.6 years. Among the IIM patients, disease duration differed not significantly (median 9.2) [34].

In the quantitative studies, disease severity, functional or motor status were mainly assessed with clinical tests, e.g. Timed-up-and-go, manual and/or quantitative muscle strength testing [36, 38]. Goyal et al. used an IBM specific outcome measure for **disease severity** (IBM functional rating scale, IBMFRS [43]), identifying lower scores in patients harbouring NT5c1A antibodies, in contrast to seronegative patients; however, findings did not reach statistical significance (p = 0.06) [36]. One study surveyed disease presentation, but did not report data [34]. PROMs as Amyotrophic Lateral Sclerosis Functional Rating Scale (ALS-FRS) [44] and Health Assessment Questionnaire (HAQ) [45] measured disease severity. HAQ values differed significantly (p < 0.01) among NMD [39]. In total, IBM scored the second highest HAQ score after limb girdle muscular dystrophies (1.9 ± 0.9), also in all sub-scores except ‘Reach’ [39].

Evaluation of the motor status identified a higher symptom burden for the included NT5c1A seropositive patients, consisting of predominant weakness in the lower legs in contrast to the onset of weakness in the upper extremities or bulbar involvement [36].

### Study objectives and types of HRQoL and illness assessment

Table 2 summarizes all study objectives. The articles explored the patient reported HRQoL within different foci: persisting symptoms [35], phenotypic differences [36], clinical and demographic variables [34], disease severity, [38, 39] and perception of illness [39]. Mental illnesses were explicitly considered twice, as depression [38] or depression and anxiety [39]. One qualitative study addressed a wider focus on HRQoL and mental health aspects [37].

Table 3 and Additional file 3 show the diverse PROMs of the applied quantitative HRQoL assessments. The two qualitative interview formats range from an open approach (focus group of IIM patients with minor structured questions [37]) to a semi structured individual approach with IBM patients [35]. Findings referring to HRQoL in IBM compared to other NMD were extracted from the primary studies in Table 3 and are thematically integrated into the narrative syntheses in the next sections.

**Table 3:** Narrative summary of HRQoL findings for IBM patients

Study	HRQoL and mental illness assessments	Values compared	IBM HRQoL dimensions*		
			Physical	Psychological	Social
Feldon et al. [34]	*HRQoL* SF-12 (PCS, MCS)	Age- and sex-matched normative US sample and rheumatoid arthritis patients	*PCS:* 30 **IBM diagnosis** **IBM impacted PCS relatively to DM/PM:** **ß -8.94 ± 0.80, p < 0.001** **Effect on work negative effect of IBM** **on work performance impacted PCS negatively: **− **2.82 ± 0.83, p < 0.001** Treated by rheumatologist negative effect on PCS: − 1.22 ± 0.81, p = 0.133 **Joint swelling negative effect on PCS: **− **1.75 ± 0.80, p = 0.029** **Multiple immunomodulators negative effect on PCS: **− **1.79 ± 0.82, p = 0.029** Lung disease negative effect on PCS: − 0.73 ± 0.92, p = 0.428;	*MCS*: 46.6 IBM diagnosis no difference among IIM; IBM impacts MCS not differently relatively to DM/PM: ß − 1.10 ± 0.83, p = 0.189 Disease duration positive effect on MCS: 0.14 ± 0.06, p = 0.233 **Effect on work negative effect of IBM on work performance impacted MCS negatively: **− **2.82 ± 1.40, p = 0.044** **Treated by rheumatologist negative effect on MCS: **− **3.00 ± 1.33, p = 0.025** **Dysphagia negative effect on MCS: **− **2.30 ± 1.16, p = 0.048** Lung disease negative effect on MCS: − 2.80 ± 1.57, p = 0.076	
Goyal et al. [36]	*HRQoL* EQ-5D-5L, EQ VAS	*NT5c1A antibodies*	Median seropositive: 55 (25–80), seronegative: 65 (50- 80); no difference (p = 0.14) among seropositive or seronegative patients		
Rose et al. [39]	*HRQoL* SF-36	*Normal population (data not shown)*	*Reduction of physical domains* **Reduction of ‘Physical Functioning’, ‘Role Physical’ and ‘General Health’ compared to values of normal population;**	*Reduction of mental health domains*	*Reduction of social domains*
	*HRQoL* INQoL	*NMD (cf. Table **1**)*	*Reduction of HRQOL*		
			Weakness 64.2 ± 28.4, Pain 46.0 ± 29.3, Locking 30.9 ± 27.3, Fatigue 54.9 ± 25.7	Emotional 40.6 ± 23.4, body image 55.5 ± 28.6;	Activity 58.0 ± 24.0, Independence 55.1 ± 33.4, Social 32.8 ± 26.7 **Intergroup differences between NMD: ‘Independence’ (ANOVA F 5.2; p < 0.001)** **‘Activity’ (ANOVA F 5.5; p < 0.001)**
	*Anxiety and depression* HADS		Depression mainly affected ‘Fatigue’	No significant differences among NMD between HADS anxiety (F 2.90; P 0.01) and depression (F 0.4; P 0.86) Depression mainly affected ‘Emotional’;	Depression mainly affected ‘Social’
Sadjadi et al. [38]	*HRQoL* SF-36V.1	*US normal population (Z scores)*	*Reduction of all physical domains* **Physical Functioning 24.21 ± 19.59, difference relatively to FSHD, MD, CMT1** **Role Physical 38.75 ± 41.02, difference relatively to NMD** Bodily pain 68.61 ± 27.17 **General Health 57.69 ± 20.67, difference relatively to NMD, MD; Vitality score 47.06 ± 21.27** Strong correlation between MMT, timed stand, time walk and ALS-FRS and ‘Physical Functioning’; Moderate correlation between ALS-FRS and ‘Role Physical’ and ‘Vitality’;	*No reduction of mental health domains* Role-emotional 75.71 ± 37.05 **Mental health 78.34 ± 15.68** **difference relatively to NMD, CMT1** Moderate correlation between timed walk and ‘Role Emotional’	*Reduction of social domain* **Social Functioning** 66.04 ± 26.85 Moderate correlation between timed walk and ALS-FRS and ‘Social Functioning’;
			**Correlation between ALS-FRS and HRQoL**		
	*Mood/depression* BDI		Strong correlation between BDI and ‘General Health’, ‘Vitality’; Moderate correlation between BDI and ‘Physical Functioning’, ‘Role Physical’, ‘Bodily Pain’;	Strong correlation between BDI and ‘Mental Health’; Mild correlation between BDI score and ALS-FRS (− 0.32, p < 0.001); Mild correlation between ALS-FRS and BDI (correlation coefficient − 0.32, p < 0.001)	Strong correlation between BDI and ‘Social Functioning’
			Correlation between depression and HRQoL 1–14% of the correlation between disease severity and HRQoL was mediated by depression		
Gibson et al. [35]	Individual semistructured in-depth interviews**		**Physical impairments (mobility and walking, fine motor skills, weakness of shoulders and trunk muscles)** (+) Mobility and ambulation as great influence (problems with stairs, avoiding stairs, use of mobility aids, getting up from a seated position, falls); (+) Swallowing problems; (+) Disease specific impairments (specific limitations in ADL, gastrointestinal complaints, fatigue, communication problems, pain, sleep disturbances, respiratory impairment, dizziness); (−) Facial weakness (chewing for longer, use of a straw);	(+) Mental impairments; (+) Emotional distress (fear of falling, thoughts about the future); (+) Impaired body image due to decrease of muscles;	(+) Social impairments; (+) Social role dissatisfaction and limitation (reliance on family members, avoidance of social situations); (−) Hand weakness in everyday life (typing, texting, use of a telephone);
Ortega et al. [37]	Focus groups interviews**		(−) Changes of quality of life	(−) Individual, need-oriented information from physicians; (−) Discussion with physician of individual patient preferences on therapies (especially medication), self-determined use of medication and endurance of side effects; (−) Future impact of IBM on everyday life and patient-relevant activities; (−) Changes of quality of life	(−) Future impact of IBM on everyday life and patient-relevant activities;

ANOVA F: analysis of variance, F-statistic; BDI: Beck Depression Inventory; CMT1: Charcot-Marie-Tooth type 1; DM: dermatomyositis; EQ-5D-5L: 5-level EQ-5D version of European quality of life questionnaire; EQ VAS: EQ visual analogue scale, access www.euroqol.org; FSHD: facioscapulohumeral muscular dystrophy; HADS: Hospital Anxiety and Depression scale; IBM: inclusion body myositis; IIM: idiopathic inflammatory myopathies; INQoL: Individualized Neuromuscular Quality of Life questionnaire [47]; MCS: mental component summary, items of psychological and social health from SF-36 are aggregated to MCS; MD: myotonic dystrophy; PCS: physical component summary; SF-12: 12-Item Short-Form Health Survey SF-36: 36-Item Short-Form Health Survey;

*Data are reported narratively. All values are shown, if data were reported in the included studies. Values are reported as mean ± SD or (range or IQR)

**Statements for which statistically significant data were shown or a “high confidence” was assessed according to CERQual are marked in bolt type. “Moderate”, “low” or “very low” assessed confidence are marked respectively with (+), (−), (−). Shown qualitative findings correspond to the summaries of review findings

### To what extent does IBM influence the HRQoL dimensions?

Quantitative and qualitative findings were narratively summarized, contrasted and categorized to the three HRQoL dimensions (Table 3). One total value of the EQ-5D-5L could not be allocated to the HRQoL dimensions. No significant difference (p = 0.14) was identified between patients harbouring NT5c1A antibodies (total value EQ-5D-5L: 55, range 25–80) and seronegative patients (total value EQ-5D-5L: 65, range 50- 80) [36].

Significant correlations were reported for disease severity (ALS-FRS) and HRQoL (SF-36) in IBM patients [38], and partially strong and moderate correlations in a group of patients with different NMD for some HRQoL domains, applying HAQ, Individualized Neuromuscular Quality of Life Questionnaire (INQoL) and SF-36 (data not shown) [39]. However, the role of age and disease severity was inconsistent among IIM and NMD [34, 38, 39]. Further, patients perceived possible changes of their HRQoL due to IBM as important in a qualitative study [37].

The geographic region of residence was not associated as a determinant of HRQoL [34].

#### Determinants of physical HRQoL

All studies identified the physical dimension as severely impaired. Among IIM, the **diagnosis of IBM** significantly impacted physical HRQoL [34]. Except for ‘Pain’, all reductions in the physical domains (SF-12) were significant for IBM: ‘Physical Functioning’, ‘Role Physical’, ‘General Health’ and ‘Vitality’, whereas disease severity correlated moderately with ‘Vitality’ and ‘Role Physical’ [38]. Aggregating IBM patients with other NMD, significant reductions were observed in ‘Physical Functioning’, ‘Role Physical’ and ‘General Health’ compared to a healthy population [39].

With a “high confidence” according to the GRADE CERQual checklist, the qualitative results reported major impairments in **ambulation** and **mobility**, especially while walking, climbing stairs or getting up from sitting position. Additional to the **weakness** of the lower extremities, weakness in trunk and shoulders were perceived. Patients adjusted their behaviour by using assistive mobility devices and avoiding stairs [35]. IBM patients scored highest in the INQoL domains ‘Weakness’ and lowest in ‘Locking’, compared to other NMD patients [39]. The differences in ‘Weakness’ among the values for NMD patients were thereby explained by 33% (p < 0.01) with disease severity (HAQ) and additional 12% with the illness perceptions (measured with the Illness Perception Questionnaire, IPQ-R [48]), considering significantly different HAQ values between the NMD [39]. Strong correlations were identified between ‘Fatigue’ and the physical component summary (PCS), ‘Weakness’ and PCS as well as HAQ, in contrast to mild correlations between HAQ and ‘Fatigue’ [39].

Strong correlations were also observed between clinical measures as MMT, timed stand, timed walk or disease severity, and ‘Physical Functioning’ [38]. Interestingly, disease duration did not significantly impact or correlate with PCS [34, 38]. Depression correlated strongly with ‘General Health’ and ‘Vitality’, and moderately with ‘Physical Functioning’, ‘Bodily Pain’ and ‘Role Physical’ [38].

Dysphagia and **specific impairments** such as pain, sleep disturbance, fatigue, or gastrointestinal problems decreased the perceived physical HRQoL [35], but minor intergroup differences were shown for INQoL symptom impact scores in NMD [39]. A significant reduction in physical HRQoL was found for concomitant joint swelling [34]. For facial weakness and adjustments in eating (longer duration, assistive devices), the confidence of the qualitative findings was “low” according to GRADE CERQual [35]. A negative physical effect was identified for IBM patients with a lung disease [34].

Medication with **multiple immunomodulators** showed a significant negative impact on physical HRQoL, as well as patients, who perceived limitations of their **work performance** due to IBM [34]. Of note, treatment by rheumatologists impacted physical HRQoL negatively [34]. Anxiety and depression correlated moderately with most physical INQoL scores in NMD [39].

#### Determinants of psychological HRQoL

The reports varied regarding psychological HRQoL. Including IBM into a group of NMD, ‘Mental Health’ was either negatively impaired without statistical significance [39] or even not reduced [38]. Feldon et al. [34] identified no significant differences of the ‘Mental Health’ scores (SF-12) in IIM patients, but disease duration positively impacted the mental component summary (MCS) in IBM patients. ‘Role Emotional’ was once moderately correlated with the timed walk [38]. Further, ‘Emotional’ strongly correlated with anxiety and depression, and with the IPQ-R domains ‘Identity’ and ‘Consequences’ [39].

Qualitatively reported psychological impairments with a “moderate confidence” according to GRADE CERQual—mainly emotional distress and impaired body image—were supported by quantitative data [35, 39]. The depression values of the Beck Depression Inventory (BDI) were strongly correlated with ‘Mental health’ (SF-36), whereas the BDI correlated mildly (− 0.32, p > 0.001) with disease severity (ALS-FRS) [38]. Among NMD, no intergroup differences were observed between the values of the Hospital Anxiety and Depression Scale (HADS) [39]. The differences in the INQoL ‘Body Image’ and ‘Emotional’ were hereby explained with 53% from mood (HADS) and with 49% from illness perceptions (IPQ-R) in NMD patients [39].

Physical symptoms such as **dysphagia** and lung disease effected psychological HRQoL negatively, significantly for the former [34].

Further, significant negative effects were reported if patients were **treated by a rheumatologist** and experienced a **limited work performance** [34]**.** “Low confidence” according to GRADE CERQual was assessed to the following qualitative findings: psychological HRQoL is affected if the patient-physician relationship is not individualised, and preferences regarding treatment options are not considered; HRQoL changes and activities of daily living affect the psychological dimension [37].

#### Determinants of social HRQoL

Data on the social HRQoL were sparse. ‘**Social Functioning**’ (SF-36) was significantly reduced in an IBM patient group [38]. However, in a group with various NMD, the scales for the ‘Social’ domain were also negatively affected, but not significant compared to the normal population [39]. Social Functioning correlated moderately with timed walk and disease severity (ALS-FRS) [38]. The main predictor of ‘Activity’ and ‘Independence’ in a NMD patient group was **disease severity** (HAQ), contributing approximately 55% to the respective scores, showing strong correlations [39]. Additional 6% contributed to the patients’ illness perceptions (IPQ-R) to the variance of ‘Independence’ [39]. The INQoL ‘Social’ domain was mainly predicted by **mood** (HADS) (45%) and **illness perception** (43%) of NMD patients [39]. A strong correlation between depression (BDI) and ‘Social Functioning’ in an IBM patient group [38] was similarly identified for the depression values of the HADS in the NMD group [39]. Relatively to these NMD, IBM scaled highest in ‘Social’ as assessed with the INQoL [39]. Qualitative findings with “moderate confidence” according to GRADE CERQual substantiated social impairments, dissatisfactions regarding social role and respective limitations. Behavioural adjustments were mainly made to avoid social events and to consider familiar support [35].

A “very low confidence” according to GRADE CERQual yielded the qualitative statement that IBM impacted everyday life like activities and communication (texting, typing) due to hand muscle weakness [35, 37]. Therefore, NMD were adversely affected regarding ‘Independence’ and ‘Activity’ with significant intergroup differences [39].

#### Interrelationship of mental illnesses and HRQoL

**Depression** as a mental illness was investigated, but the role of mental illness relating to HRQoL was only measured in two studies [38, 39]. One study identified that HRQoL and disease severity are significantly correlated, and that the values for depression (BDI) also correlated with disease severity and HRQoL. Additionally, depression as a mediator reduced the correlation between HRQoL and disease severity of 1–14% [38]. Furthermore, the total INQoL scale showed a moderate correlation (p > 0.01) with **anxiety and depression** (HADS) in NMD patients [39].

## Discussion

To summarize, the literature was sparse regarding social and psychological HRQoL as well as mental health in IBM. The impaired physical HRQoL was most evident in relation to the general population or other NMD. In interpreting these findings, we need to consider that the qualitative data increase the understanding of the quantitative data. Relevant determinants for a comprehensive understanding of the patient relevant symptom-HRQoL interplay could be illustrated exploratively. Patients’ weakness and swallowing problems as well as decreased functioning and role perceptions specify physical HRQoL. Practical support in form of mobility devices or from family members appear relevant to maintain social and psychological HRQoL despite physical vulnerability.

Our findings suggest that the results for psychological and social HRQoL are less applicable than for physical HRQoL. One key problem is the variability of the applied PROMs. Therefore, comparison of the scarce values is difficult, if recommended outcome assessments have not yet been widely implemented and harmonized [49]. Unfortunately, the aggregated values for IIM or NMD groups diluted the specific evidence for IBM [38]. At this point, we should not jump to conclusions on the cause of determinants of HRQoL in IBM while data are not fully reported or the study designs are mainly cross-sectional. According to our findings, IBM patients seem to be only mildly impaired regarding their psychological and social HRQoL as well as mental health in contrast to other NMD patients. However, mental illnesses like depression or anxiety might play an important role as a mediator in the evaluation of holistic HRQoL.

### Risk factors and critical events along the patient journey

Previous research suggest that neither age, disease duration nor disease severity are evident risk factors for a decreased HRQoL in NMD patients, and thus for IBM [10, 11, 17]. Hence, the six studies included in our review did not broadly report on the actual care situation, socioeconomic characteristics or marital status determining HRQoL. Suzuki et al. [50] would be a good example for a more holistic approach to data collection on the IBM care situation and natural history of the disease. However, comparison of this data with the reviewed studies is difficult. It would be valuable to integrate established HRQoL and mental health measurements in such long-term IBM studies.

Established physical milestones in the IBM patient journey concentrate on functional or clinical endpoints [21, 50]. Surprisingly, falls were only mentioned in one qualitative study, whereas dysphagia has been considered to a larger extent in the other studies included in this review. Until now, traditional approaches have failed to identify further milestones of social and psychological HRQoL, which could more precisely illustrate the disease burden during progression. As long as IBM is largely refractory to treatment, social or psychological limitations could then be antagonized with tailored interventions. Future mental illnesses or social isolation might thereby prevented or delayed, especially in an older patient group [51–53]. The described relevance of employment status and attending healthcare providers might indicate a need to consider even more individual patient characteristics in the long-term decision making for such supportive therapies [54].

### Future challenges for research investigations

An extensive comparison of how IBM influences HRQoL or mental health in contrast to other NMD was not possible in our systematic review. Primary research is needed as ground for future comparisons. It is challenging to choose suitable clinical endpoints in clinical trials or health technology assessments, especially PROMs in orphan diseases such as IBM [55–57]. Generally, the use of PROMs is rather scarce in orphan drug labels that are approved by the US Food and Drug Administration [58]. Current trials with Arimoclomol and Sirolimus applied the IBMFRS as primary outcome measure (ClinicalTrials.gov Identifiers: NCT04049097, NCT04789070). Although the IBMFRS is clinician-administered, it comprises relevant aspects of daily living with IBM [43, 49]. Considerably, more work about the role of mental illnesses in IBM patients, their risk factors but also protective factors could be useful to fully understand the determinants of HRQoL during progression. Therefore, pragmatic mixed methods approaches could not only enhance a patient-focused orphan drug development, but also evaluate supportive therapies effectively to raise or maintain HRQoL of patients and their families [59].

If the body of knowledge is non-existent or sparse, qualitative interviews could open new ways to develop concepts and obtain a deeper understanding of a sociological phenomenon of interest in medical settings, in this case patient relevant determinants and dimensions of HRQoL in IBM [60, 61]. A study proposal for an in-depth exploratory interview study could address the research focus of describing and exploring HRQoL in IBM. In-depth interview techniques aim at eliciting extensive perspectives of the individual participants. To ensure similarity, the sample of IBM patients should meet established IBM diagnostic criteria. The underlying paradigmatic assumptions could be deductively derived from existing knowledge about generic and specific HRQoL dimensions in NMD and IBM, as suggested in this review. It could be valuable to collect data inductively due to open questions in the interview guide. Field investigations in the actual care settings with IBM patients representing different disease characteristics (e.g. disease duration, disease severity) could be helpful to contrast the cases and better understand the meaning of relevant HRQoL dimensions and determinants. The use of researcher triangulation (e.g. medical scientists and sociologists) for data collection and analysis could strengthen the epistemological standards [62].

### Limitations

Some limitations must be addressed: an expansion of the search terms and inclusion criteria, which comprised aggregated results of IIM or NMD patient groups, might have identified more studies. So far, there are no robust data substantiating similar HRQoL changes and determinants in the IIM disease group to draw clear conclusions for IBM patients [17]. One included study exemplified significant HRQoL differences in IIM [34]. On the contrary, other studies in IIM, in which IBM is mainly not included, negate such differences [63–66]. The distinct outcome assessments, settings and identified risk of bias might further limit the results. We rated the study of Rose et al. [39] as a cross-sectional study, while the systematic review of LeClair listed it as a RCT [17]. In our opinion, the examined RCT data, the exploratory and retrospective analysis without follow-up could justify our lower rated evidence level; after several vain requests to the authors, no further unpublished data could complement the results.

## Conclusion

In conclusion, six studies reported on determinants and dimensions of HRQoL and mental illnesses in IBM in this systematic review, supporting decreased physical HRQoL in contrast to the normal population or other NMD patients. Unfortunately, rigour determinants and dimensions of HRQoL and mental illness could not be definitively clarified for IBM from the included studies. Importance is especially attributed to weakness, physical role perceptions and functioning as well as dysphagia. A research gap was identified for psychological and social HRQoL in IBM patients, although qualitative studies suggested relevant social and psychological factors for patients and caregivers. Interestingly, quantitative studies report differing values for patients’ mental health and point out a considerable role of depression as a possible mediator for HRQoL. However, qualitative in-depth studies of HRQoL and its determinants are missing until now. Our work suggests that a more holistic understanding of HRQoL in IBM is needed to identify disease specific determinants of HRQoL. Until the physical limitations in IBM cannot be cured or significantly improved, the focus should be pointed on psychosocial prevention of mental illness and support for the daily life of patients and families.

## Supplementary Information

**Additional file 1.** Search strategy.pdf. Detailed search terms of the systematic search.

**Additional file 2.** Qualitative evidence profiles.xlsx. Summaries of review findings and ratings of the qualitative studies according to GRADE CERQual.

**Additional file 3.** Overview PROMs for HRQoL assessment IBM.pdf. Overview of applied PROMs for HRQoL assessment in the included quantitative studies.

## Data Availability

The datasets supporting the conclusions of this article are included within the article and its additional files.

## Abbreviations

ALS-FRS: Amyotrophic Lateral Sclerosis Functional Rating Scale
AXIS: Appraisal tool for Cross-Sectional Studies
BDI: Beck Depression Inventory
DM: Dermatomyositis
HADS: Hospital Anxiety and Depression Scale
HAQ: Health Assessment Questionnaire
HRQoL: Health-related quality of life
IBM: Inclusion body myositis
IBMFRS: IBM Functional Rating Scale
IIM: Idiopathic inflammatory myopathies
INQoL: Individualized Neuromuscular Quality of Life Questionnaire
IPQ-R: Revised Illness Perception Questionnaire
MCS: Mental component summary
MMAT: Mixed Methods Appraisal Tool
NMD: Neuromuscular diseases
NOS: Newcastle-Ottawa scale
PCS: Physical component summary
PM: Polymyositis
PROMs: Patient reported outcome measure
