# Supplementary material for: The health-related quality of life, mental health and mental illnesses of patients with inclusion body myositis (IBM): results of a mixed methods systematic review
Source: Orphanet J Rare Dis. 2022 Jun 16;17:227. doi: 10.1186/s13023-022-02382-x (PMC9204871; doi:10.1186/s13023-022-02382-x)
Supplement: Supplementary file 1 — Additional file 1. Search strategy.pdf. Detailed search terms of the systematic search. [file 13023_2022_2382_MOESM1_ESM.pdf]

Additional file 1: Search strategy

All searches were performed on 11 February 2021

Medline (via PubMed)

#1	quality of life[MeSH Terms]	204.260
#2	"quality of life"	352.011
#3	mental disease[MeSH Terms]	1.269.220
#4	"mental disease"	1.414
#5	mental illness[MeSH Terms]	1.269.220
#6	"mental illness"	34.048
#7	mental disorder[MeSH Terms]	1.269.220
#8	"mental disorder"	9.934
#9	mental health[MeSH Terms]	41.600
#10	"mental health"	299.249
#11	well being[MeSH Terms]	378.244
#12	"well being"	85.205
#13	psychiatr*	860.778
#14	psychosocial	112.707
#15	psychologic*	661.761
#16	#1 -#15 OR	2.808.473
#17	inflammatory myopathy[MeSH Terms]	20.072
#18	"inflammatory myopathy"	1.578
#19	inflammatory muscle disease[MeSH Terms]	20.072
#20	"inflammatory muscle disease"	137
#21	idiopathic inflammatory myopathy[MeSH Terms]	20.072
#22	"idiopathic inflammatory myopathy"	571
#23	inclusion body myositis[MeSH Terms]	1.146
#24	"inclusion body myositis"	1.952
#25	Myositis[MeSH Terms]	20.072
#26	"myositis"	15.993
#27	#17 - #26 OR	24.043
#28	#16 AND #27	904
#30	Filter English	752
#31	Filter English; German	816
#32	Filter English; German; Humans	750

"((((((((((((((quality of life[MeSH Terms]) OR (""quality of life"")) OR (mental disease[MeSH Terms])) OR (""mental disease"")) OR (mental illness[MeSH Terms])) OR (""mental illness"")) OR (mental disorder[MeSH Terms])) OR (""mental disorder"")) OR (mental health[MeSH Terms])) OR (""mental health"")) OR (well being[MeSH Terms])) OR (""well being"")) OR (psychiatr*)) OR (psychosocial)) OR (psychologic*)) AND ((((((((((inflammatory myopathy[MeSH Terms]) OR (""inflammatory myopathy"")) OR (inflammatory muscle disease[MeSH Terms])) OR (""inflammatory muscle disease"")) OR (idiopathic inflammatory myopathy[MeSH Terms])) OR (""idiopathic inflammatory myopathy"")) OR (inclusion body myositis[MeSH Terms])) OR (""inclusion body myositis"")) OR (Myositis[MeSH Terms])) OR (""Myositis""))",Publication Date,"English, German, Humans","((""quality of life""[MeSH Terms] OR ""quality of life""[All Fields] OR ""mental

disorders""[MeSH Terms] OR ""mental disease""[All Fields] OR ""mental disorders""[MeSH Terms] OR ""mental illness""[All Fields] OR ""mental disorders""[MeSH Terms] OR ""mental disorder""[All Fields] OR ""mental health""[MeSH Terms] OR ""mental health""[All Fields] OR ""health""[MeSH Terms] OR ""well being""[All Fields] OR ""psychiatr*""[All Fields] OR (""psychosocial""[All Fields] OR ""psychosocially""[All Fields]) OR ""psychologic*""[All Fields]) AND (""Myositis""[MeSH Terms] OR ""inflammatory myopathy""[All Fields] OR ""Myositis""[MeSH Terms] OR ""inflammatory muscle disease""[All Fields] OR ""Myositis""[MeSH Terms] OR ""idiopathic inflammatory myopathy""[All Fields] OR ""myositis, inclusion body""[MeSH Terms] OR ""inclusion body myositis""[All Fields] OR ""Myositis""[MeSH Terms] OR ""Myositis""[All Fields])) AND ((humans[Filter]) AND (english[Filter] OR german[Filter]))",750,05:52:57

PsycINFO

#4	quality of life.mp. or exp "Quality of Life"/	92.000
#5	mental disease.mp.	2.202
#6	mental illness.mp.	45.300
#7	mental disorder.mp. or exp Mental Disorders/	881.474
#8	mental health.mp. or exp Mental Health/	228.977
#9	well being.mp. or exp Well Being/	96.494
#10	psychiatr*.mp.	341.680
#11	psychosocial.mp. or exp Psychosocial Assessment/ or exp Psychosocial Outcomes/ or exp Psychosocial Factors/	212.995
#12	exp Psychology/ or exp Psychological Assessment/ or psychologic*.mp.	675.008
#13	4 or 5 or 6 or 7 or 8 or 9 or 10 or 11 or 12	1.811.607
#1	(inclusion body myositis or myositis).mp. [mp=title, abstract, heading word, table of contents, key concepts, original title, tests & measures, mesh]	350
#2	(inflammatory myopathy or inflammatory muscle disease or idiopathic inflammatory myopathy or myositis or inclusion body myositis).mp. [mp=title, abstract, heading word, table of contents, key concepts, original title, tests & measures, mesh]	375
#3	#1 OR #2	375
#14	#3 AND #13	62
#15	limit 15 to ((english or german) and human)	52

LIVIVO

#1	"quality of life"	411.303
#2	"mental disease"	12.631
#3	"mental illness"	37.421
#4	"mental disorder"	11.543
#5	"mental health"	288.273
#6	"well being"	9.879
#7	"psychiatr*"	1.049
#8	"psychosocial"	138.049
#9	psychologic*	10.188
#10	#1 OR #2 OR #3 OR #4 OR #5 OR #6 OR #7 OR #8 OR #9	830.313
#11	"inflammatory myopathy"	1.635
#12	"inflammatory muscle disease"	170

#13	"idiopathic inflammatory myopathy"	566
#14	"inclusion body myositis"	1.662
#15	"myositis"	17.947
#16	#11 OR #12 OR #13 OR #14 OR #15	18.627
#17	(#10) AND (#16)	192
#18	#Filter Human	
#19	#17 English	174
#20	#17 German	5
#21	17 English, German	179

Cochrane

#1	"quality of life"	117.077
#2	quality of life[MeSH]	24.393
#3	"mental disease"	4.807
#4	"mental illness"	4.206
#5	mental disorder[MeSH]	73.817
#6	"mental disorder"	1.286
#7	mental health[MeSH]	1.534
#8	"mental health"	26.603
#9	"well being"	15.229
#10	psychiatr*	65.723
#11	psychosocial	17.715
#12	psychologic*	55.673
#13	#1 OR #2 OR #3 OR #4 OR #5 OR #6 OR #7 OR #8 OR #9 OR #10 OR #11 OR #12	269.334
#14	"inflammatory myopathy"	40
#15	"inflammatory muscle disease"	4
#16	"idiopathic inflammatory myopathy"	24
#17	"inclusion body myositis"	94
#18	Myositis[MeSH]	197
#19	"myositis"	532
#20	#14 OR #15 OR #16 OR #17 OR #18 OR #19	541
#21	#13 AND #20	81
