# Supplementary material for: The health-related quality of life, mental health and mental illnesses of patients with inclusion body myositis (IBM): results of a mixed methods systematic review
Source: Orphanet J Rare Dis. 2022 Jun 16;17:227. doi: 10.1186/s13023-022-02382-x (PMC9204871; doi:10.1186/s13023-022-02382-x)
Supplement: Supplementary file 3 — Additional file 3. Overview PROMs for HRQoL assessment IBM.pdf. Overview of applied PROMs for HRQoL assessment in the included quantitative studies. [file 13023_2022_2382_MOESM3_ESM.pdf]

**Overview PROMs HRQoL assessment IBM**

Overview of applied PROMs for HRQoL assessment in the included quantitative studies

| | Domains | Interpretation | Recommended by IMACS* |
|---|---|---|---|
| **Generic subjective HRQoL** | | | |
| 36-Item Short-Form Health Survey (SF-36) | Physical functioning<br>Role physical<br>Bodily pain<br>General health<br>(=Physical Health Score, PCS)<br>Vitality<br>Social functioning<br>Role-emotional<br>Mental health<br>(=Mental Health Score, MCS) | Range: 0-100 in two sub-scores (PCS, MCS);<br>100 is the best possible health status | x |
| 12-Item Short-Form Health Survey (SF-12) | Physical functioning<br>Role physical<br>Bodily pain<br>General health<br>(=Physical Health Score, PCS)<br>Vitality<br>Social functioning<br>Role-emotional<br>Mental health<br>(=Mental Health Score, MCS) | Range: 0-100 in two sub-scores (PCS, MCS);<br>100 is the best possible health status | |
| European quality of life 5D-5L questionnaire (EQ-5D-5L) | Mobility<br>Self-care<br>Usual activities<br>Pain / discomfort<br>Anxiety / depression | Index=1 is the best possible health status,<br>Index<0 is the worst possible health status | |
| EQ Visual analogue scale (VAS) | | Range 0-100 in one total score;<br>100 is the best possible health status | |

| | Domains | Interpretation | Recommended by IMACS* |
|---|---|---|---|
| **Disease specific subjective HRQoL** | | | |
| Individualized Neuromuscular Quality of Life questionnaire (INQoL) | Fatigue<br>Weakness<br>Pain<br>Locking (myotonia)<br>Emotion<br>Social<br>Independence<br>Activity<br>Body image<br>Treatment effects and expectations<br>INQoL index | Range 0-100 in 11 sub-scores and one total score;<br>Higher percentage shows a greater symptom impact, respectively a worse health status | x |
| **Subjective burden of mental illness** | | | |
| Hospital Anxiety and Depression scale (HADS) | Anxiety and depression<br>14 Items, 7 each | Range: 0-21;<br>Higher score indicates higher mental burden,<br>Serious symptoms of anxiety or depression are indicated with a sub-score >8,<br>Separate scores for depression and anxiety | |
| Beck Depression Inventory (BDI) | Depression<br>21 Items | Range 0-63;<br>Higher score indicates higher depressive burden,<br>Cut-off values:<br>0-10: no depression, clinically unremarkable or remitted<br>10-19: mild depressive syndrome<br>20-29: moderate depressive syndrome<br>≥ 30: severe depressive syndrome | |

*IMACS: International Clinical Assessment and Studies Group; Rating according to Benveniste O, Rider LG. 213th ENMC International Workshop: Outcome measures and clinical trial readiness in idiopathic inflammatory myopathies, Heemskerk, The Netherlands, 18-20 September 2015. Neuromuscul Disord. 2016;26:523–34. doi:10.1016/j.nmd.2016.05.014.
